# Salidroside alleviates cholestasis-induced liver fibrosis by inhibiting hepatic stellate cells via activation of the PI3K/AKT/GSK-3β signaling pathway and regulating intestinal flora distribution

**DOI:** 10.3389/fphar.2024.1396023

**Published:** 2024-05-14

**Authors:** Xin Wang, Shuxia Cao, Yuan Huang, Liangchang Li, Dongyuan Xu, Lan Liu

**Affiliations:** ^1^ Key Laboratory of Cellular Function and Pharmacology of Jilin Province, Yanbian University, Yanji, China; ^2^ Department of Pathology, Yanbian University Hospital, Yanji, China

**Keywords:** salidroside, cholestatic liver fibrosis, hepatic stellate cells, intestinal flora, PI3K/Akt/GSK-3β pathway

## Abstract

Salidroside (SAL), a phenylpropanoid bioactive compound, has various pharmacological properties, including antioxidant, anti-inflammatory, and hepatoprotective effects. However, the pharmacological effects and mechanisms of action of SAL on cholestatic liver injury are unclear. This study investigated the mechanism and effects of salidroside (SAL) on intestinal flora distribution and hepatic stellate cell (HSC) activation in cholestatic hepatic fibrosis. Bile duct ligation was used to cause cholestasis BALB/c mice. The therapeutic efficacy of SAL in liver fibrosis was assessed via serum/tissue biochemical analyses and liver tissue hematoxylin and eosin and Masson staining. Inflammation and oxidative stress were analyzed using enzyme-linked immunosorbent assay and western blotting. HSC were activated *in vitro* using lipopolysaccharide, and the effects of SAL on HSC migration and inflammatory factor expression were detected via scratch, transwell, and western blotting assays. The effects of SAL on the PI3K/AKT/GSK-3β pathway *in vivo* and *in vitro* were detected using western blotting. 16sRNA sequencing was used to detect the effect of SAL on the diversity of the intestinal flora. Ileal histopathology and western blotting were used to detect the protective effect of SAL on the intestinal mucosal barrier. SAL reduces liver inflammation and oxidative stress and protects against liver fibrosis with cholestasis. It inhibits HSC activation and activates the PI3K/AKT/GSK-3β pathway *in vitro* and *in vivo*. Additionally, SAL restores the abundance of intestinal flora, which contributes to the repair of the intestinal mucosal barrier, inhibits endotoxin translocation, and indirectly inhibits HSC activation, reversing the course of cholestatic liver fibrosis. SAL inhibits HSC activation through the PI3K/AKT/GSK-3β pathway and improves intestinal flora distribution, thereby protecting and reversing the progression of hepatic fibrosis.

## Graphical Abstract

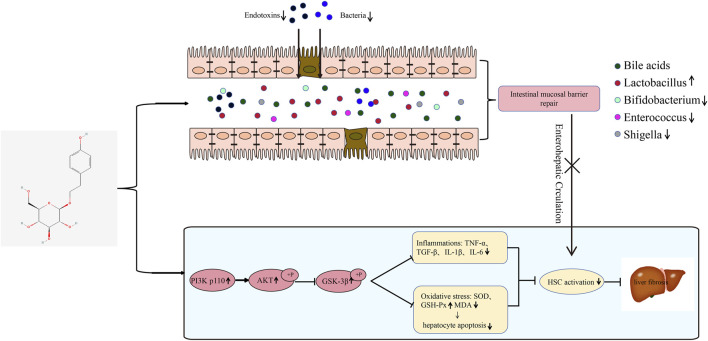

## 1 Introduction

Cholestatic liver fibrosis is a common liver injury and is typically caused by drug toxicity, inflammatory response, and biliary tract obstruction (Yokoda and Rodriguez, 2020). When the biliary system is damaged, the slugged bile acids (BA) recruit several inflammatory cells and release considerable inflammatory factors, which leads to proliferation and activation of hepatic stellate cells (HSC), liver injury, hepatic fibrosis, and ultimately cirrhosis (Zhang et al., 2023). HSC are important in the process of hepatic fibrogenesis, and when the liver is stimulated, silent HSC are activated, causing an inflammatory response, apoptosis, necrosis, hypoxia of cell tissues, and oxidative stress. Activated HSC can produce large amounts of α-SMA and extracellular matrices (ECM) and have the ability to proliferate and migrate, further contributing to hepatic inflammation (Guo and Friedman, 2007). Therefore, inhibiting HSC activation and the inflammatory response may be an important strategy to prevent and improve cholestatic liver injury.

PI3K/AKT is a signaling pathway associated with inflammation, oxidative stress, and apoptosis and plays an important role in a variety of physiological and pathological processes, including cell survival, differentiation, growth, motility, and apoptosis. This signaling pathway also regulates the development of hepatic fibrosis by participating in HSC activation (Wu L. et al., 2017). GSK-3β reportedly inhibits the expression of HSC activation-related proteins (Lee et al., 2021). GSK-3β is a downstream target of the PI3K/AKT signaling pathway, and is involved in lung, kidney, and cardiac fibrosis through this pathway (Liu et al., 2020; Hu et al., 2022). However, the role of the PI3K/AKT/GSK-3β signaling pathway in cholestatic liver fibrosis remains unclarified.

The intestines are anatomically closely related to the liver, and more than half of the blood in the portal vein is supplied by the mesenteric vein (Henao-Mejia et al., 2013). Due to the presence of the liver-gut axis, the liver and intestines interact with each other through a variety of physiological activities. Hence, an imbalance in intestinal flora homeostasis is strongly associated with liver injury due to cholestasis (Kummen et al., 2017). Disorders of intestinal bacteria cause changes in the permeability of the intestinal mucosa, allowing for toxic substances to penetrate the intestinal mucosal barrier and enter the liver through the bloodstream. Intrinsic immune cells and HSC in the liver participate in the inflammatory response induced by pattern recognition receptor interactions, exacerbating liver inflammation and fibrosis.

Salidroside (SAL), a phenylpropanoid bioactive compound, has various pharmacological properties, including antioxidant (Chen et al., 2009), anti-inflammatory (Guan et al., 2012), and hepatoprotective effects (Zheng et al., 2018). Recently, it has been found that SAL can attenuate carbon tetrachloride-induced hepatic fibrosis by inhibiting HSC activation through the upregulation of miRNA and downregulation of JNK expression (Ye et al., 2021; Lang et al., 2023). In addition, SAL ameliorates hepatic fibrosis in mice with non-alcoholic steatohepatitis by suppressing HSC activation via the MAPK signaling pathway inhibition and by inhibiting oxidative stress and oxygen radical production (Yang et al., 2016; Hu et al., 2021). However, the pharmacological effects and mechanisms of action of SAL on cholestatic liver injury are unclear. As the PI3K/AKT/GSK-3β signaling pathway is closely related to the development of liver fibrosis, this study explored the effects of SAL in bile duct ligation-induced cholestatic liver fibrosis using *in vivo* experiments. Further, the changes in the microflora of the feces were detected, and the mechanism of SAL in alleviating liver fibrosis was further explored *in vitro*. The anti-fibrosis role of SAL via regulation of the PI3K/AKT/GSK-3β signaling pathway was also investigated.

## 2 Methods and materials

### 2.1 Preparation of animal models

The BALB/c mice (male, 8 weeks old) were purchased from the Animal Experiment Centre of Yanbian University. All animal experiments were performed following the ARRIVE guidelines and were approved by the Animal Ethics Committee of Yanbian University (Approval No. YD202203012001). All mice were acclimatized for 1 week before the experiment. Each mouse was allowed access to food and water *ad libitum*, and the indoor temperature was maintained at approximately 25°C with relative humidity at 55%. The mice were anaesthetized with 2% isoflurane, the bile ducts were isolated, the proximal and distal segments of the bile ducts were ligated, and the bile ducts were transected between the sutures. The abdominal organs were returned, and the peritoneum and skin were sutured in layers.

### 2.2 Experimental program

BALB/c mice were randomly divided into 5 groups (*n* = 8): control group (CON), bile duct ligation model group (MOD), SAL (Solarbio, Beijing, China) low-dose group (L), SAL medium-dose group (M), and SAL high-dose group (H). The CON group of mice underwent laparotomy, without bile duct ligation. For the CON and MOD groups, the mice were administered 0.9% normal saline. Mice were administered 5 mg/kg SAL by gavage in the low-dose group after bile duct ligation, 10 mg/kg SAL by gavage in the medium-dose group, and 20 mg/kg SAL by gavage in the high-dose group. The SAL was administered for 2 weeks, starting on the fourth day after the bile duct ligation.

### 2.3 Cell culture

Hepatic stellate cells (HSCs: LX-2 derived from human hepatic stellate cells) were obtained from the cell bank of the Typical Culture Preservation Committee of the Chinese Academy of Sciences. Cell culture was performed in strict accordance with the HSC. HSCs were cultured in Dulbecco’s modified Eagle’s medium (DMEM Gibco, NY, United States) supplemented with 1% Penicillin–Streptomycin Liquid (Solarbio) and 10% fetal bovine serum (VivaCell, Shanghai, China). Lipopolysaccharide (LPS; Sigma-Aldrich United States) was used to activate HSCs in culture.

### 2.4 Histopathological analysis

Liver and terminal ileum tissues were collected and fixed in 10% formalin solution. The tissues were embedded in paraffin after dehydration in a graded series of ethyl alcohol. Tissue specimens were stained with hematoxylin and eosin (H&E) (Solarbio), Masson (Solarbio) and periodic acid-Schiff (PAS) (Solarbio) according to the instructions. Each tissue sample was observed using light microscopy (Nikon, Japan).

### 2.5 Immunohistochemical staining

The tissues were embedded in paraffin after dehydration in a graded series of ethyl alcohol. Sodium citrate (Solarbio) was used for antigen repair before being washed in PBS (Solarbio). The primary antibodies PI3K p110 (1:100), p-AKT (1:100), and p-GSK-3β (1:100) were incubated overnight at 4°C in the refrigerator (for specific information on antibodies, please refer to Supplementary Data S1). Each tissue sample was observed using light microscopy (Nikon).

### 2.6 Enzyme-linked immunosorbent assay (ELISA)

The blood was collected and centrifuged (3,500 rpm/min for 10 min at 4°C), and the upper serum layer was collected. The expression levels of TNF-α, TGF-β, IL-1β, and IL-6 in mice were detected with ELISA kits (mlbio, Shanghai, China) by strictly following the instructions provided.

### 2.7 Total bile acid test

The total bile acid (TBA) in serum and the liver were determined by ELISA. Blood samples were placed at room temperature for 30 min, centrifuged (3,500 rpm/min for 10 min at 4°C) to obtain the serum, and then stored at −80°C. Approximately 100 mg of liver tissue was placed in 0.9% normal saline, homogenized by a tissue homogenizer, centrifuged at 3,500 rpm/min for 10 min, and the supernatant was obtained. The TBA level in the supernatant was determined according to the instructions of the commercial kits (mlbio). The total protein in the liver was detected via the BCA method using a Pierce BCA protein Assay Kit (Thermo Fisher Scientific, MA, United States). TBA was normalized with total protein.

### 2.8 Liver oxidative stress indicator assay

Livers were isolated from mice, washed with saline, and put into EP tubes. The EP tube was filled with saline equivalent to 9 times the weight of the liver, and it was mashed with a homogenizer before being centrifuged at 3,500 rpm for 10 min to collect the supernatant. The degree of hepatic oxidative stress was detected according to the instructions of the Superoxide Dismutase (SOD) Kit, Malondialdehyde (MDA) Kit, and Glutathione Peroxidase (GSH-Px) Kit (Solarbio).

### 2.9 Serum biochemical analysis

Blood samples were placed at room temperature for 30 min and centrifuged at 3,500 rpm for 10 min to collect the upper serum layer. The kit instructions for alanine aminotransferase (ALT), Aspartate aminotransferase (AST), albumin (ALB), and Total bilirubin (TBIL) kits (Nanjing Jiancheng Bioengineering Institute, Nanjing, China) were strictly followed to detect the various indicators.

### 2.10 Western blotting

Isolation of cell/tissue proteins was performed using RIPA Rapid Lysate Extraction. The concentrations of the extracted proteins were determined using the BCA standard protein kit (Thermo Fisher Scientific). Sample proteins were electrophoresed by SDS-PAGE and transferred to a PVDF membrane. Skimmed milk powder closed for 1.5 h. The corresponding primary antibodies (1:1,000) were incubated, refrigerated at 4°C overnight, and developed by reacting the corresponding secondary antibodies for 1.5 h at room temperature (for specific information on antibodies, please refer to Supplementary Data S1).

### 2.11 Cellular immunofluorescence

Cells were inoculated in 6-well plates. The cells were permeabilized with 0.4% Triton, then fixed with 4% paraformaldehyde and contained with 1% BSA for 1 h. Primary antibodies were added and incubated overnight, followed by incubation with the corresponding fluorescent secondary antibodies and staining with DAPI. Photographic observation was performed using a microscope (Leica, German).

### 2.12 Detection of endotoxin, diamine oxidase (DAO) and D-lactate

Blood was collected from the mice and centrifuged at 3,500 rpm for 10 min at 4°C to collect the upper serum. The concentrations of endotoxin, DAO, and D-lactic acid (Shanghai Lengton Biotechnology Co., Ltd., China) in serum were detected in strict accordance with the kit instructions.

### 2.13 Bioinformatics

Firstly, SAL targets were screened by PubChem (https://pubchem.ncbi.nlm.nih.gov/) and SwissTargetPrediction (http://www.swisstargetprediction.ch/) databases. Next, the ParmGKB database (https://www.pharmgkb.org), the TTD database (https://db.idrblab.net/ttd/), the NCBI database (https://www.ncbi.nlm.nih.gov/), DisGeNET database (https://www.disgenet.org/), and the GeneCards database (https://www.genecards.org/) were used to screen for genes involved in the pathogenesis of cholestatic liver fibrosis. The intersecting genes between SAL targets and cholestatic liver fibrosis pathogenesis genes were then screened using the jvenn diagram viewer (Bardou et al., 2014), and the screened intersecting genes were visualized using the Cytoscape software. PPI protein network analysis of intersecting genes was conducted using the STRING database. Intersecting genes were ranked according to Betweenness Centrality using the cytoNCA plugin in the Cytoscape software to screen the top five core targets.

### 2.14 Intestinal flora test

Mice in the control, model, and high dose groups were placed in cages with clean filter paper. Mouse excreta were quickly collected after defecation, collecting 5–8 pellets of excreta from each mouse, and replacing the filter paper with a new one each time. Total DNA in the feces was extracted according to the instructions of the FastDNA^®^ Spin Kit for Soil (Omega Bio-Tek, Georgia, United States). DNA integrity was checked using 1% agarose gel electrophoresis. The concentration and purity of total DNA was measured using the NanoDrop2000 and the product was purified using the AxyPrep DNA Gel Extraction Kit (Axygen, California, United States). PCR amplification of the V3-V4 region of the 16S rRNA of bacteria in all samples of intestinal contents was performed with the upstream primer 338F: ACT​CCT​ACG​GGG​AGG​CAG​CAG and the downstream primer 806R: GGACTACHVGGGGTWTCTAAT. The PCR products from the same sample were mixed and detected by 2% agarose gel electrophoresis. The PCR products were recovered by cutting the gel using the AxyPrep DNA gel recovery kit (Axygen, California, United States) with Tris HCL eluted. Amplification was performed using transgenap221-02. PE libraries were constructed using the NEXTFLEX Rapid DNA-Seq Kit and sequenced using Illumina’s Miseq PE300. The above experiments were entrusted to Majorbio.

### 2.15 Statistical analysis

ImageJ software was used to analyze the images in the experiment. Graphpad Prism 6.02 software was used for statistical analyses in the study. The experimental data were expressed as mean ± SD, the *p*-value < 0.05 was considered with statistical significance. A *t*-test was used to compare the two groups, and differences among groups were examined by one-way ANOVA, followed by Tukey’s post-test.

## 3 Results

### 3.1 SAL reduces the inflammatory response caused by cholestasis

**FIGURE 1:**
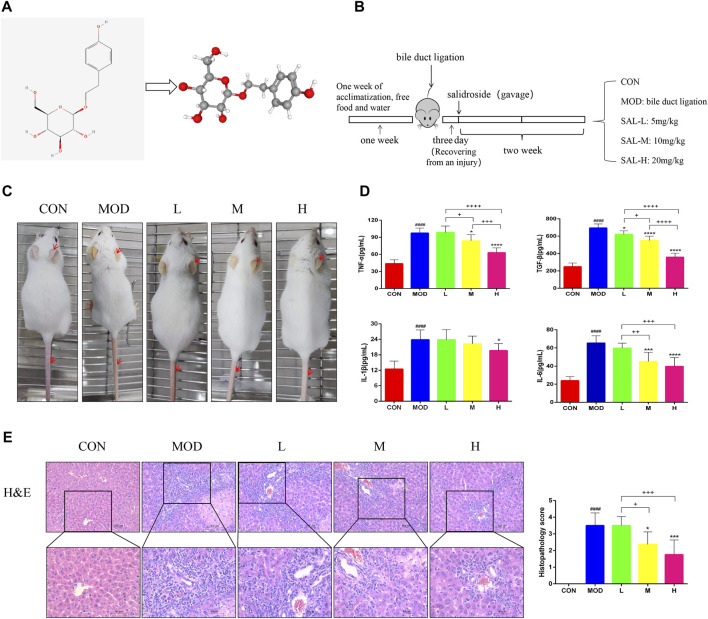
Effect of SAL on liver inflammation caused by cholestasis. **(A)** SAL structure chart. **(B)** Experimental process. **(C)** Expression of cutaneous jaundice in mice. **(D)** Expression of inflammatory factors in the mouse liver. **(E)** H&E staining of liver tissue. ^####^ *p* < 0.0001 VS. CON; *p < 0.05, ***p < 0.001, ****p < 0.0001 VS. MOD; ^+^ *p* < 0.05, ^++^ *p* < 0.01, ^+++^ *p* < 0.001, ^++++^ *p* < 0.0001. Data are expressed as mean ± SD.

The two- and three-dimensional structures of SAL are shown in Figure 1A. The experimental procedure is illustrated in Figure 1B. Mice in the model group showed significant manifestations of jaundice compared to mice in the control group; for example, the skin on the ears and tail was noticeably yellow, and the hair was dry and lacked luster (Figure 1C). The expressions of pro-inflammatory factors TNF-α, TGF-β, IL-1β, and IL-6 were considerably increased in the model group (Figure 1D). Jaundice and inflammatory manifestations were significantly reduced in mice after SAL treatment (Figures 1C, D). In addition, tissue staining showed that the normal lobular structure of the livers of bile duct-ligated mice was disrupted and a large amount of fibrous tissue had formed, accompanied by a large amount of inflammatory cell infiltration and massive necrosis of the hepatocytes. SAL treatment reduced liver fibrosis and areas of hepatocellular necrosis, especially when high-doses were administered. Small amounts of fibrous tissue and inflammatory cell infiltration were observed around the vessels (Figure 1E). These results suggest that SAL may attenuate the hepatic inflammatory response to biliary ligation.

### 3.2 SAL attenuates oxidative stress and necrosis of hepatocytes caused by cholestasis

**FIGURE 2:**
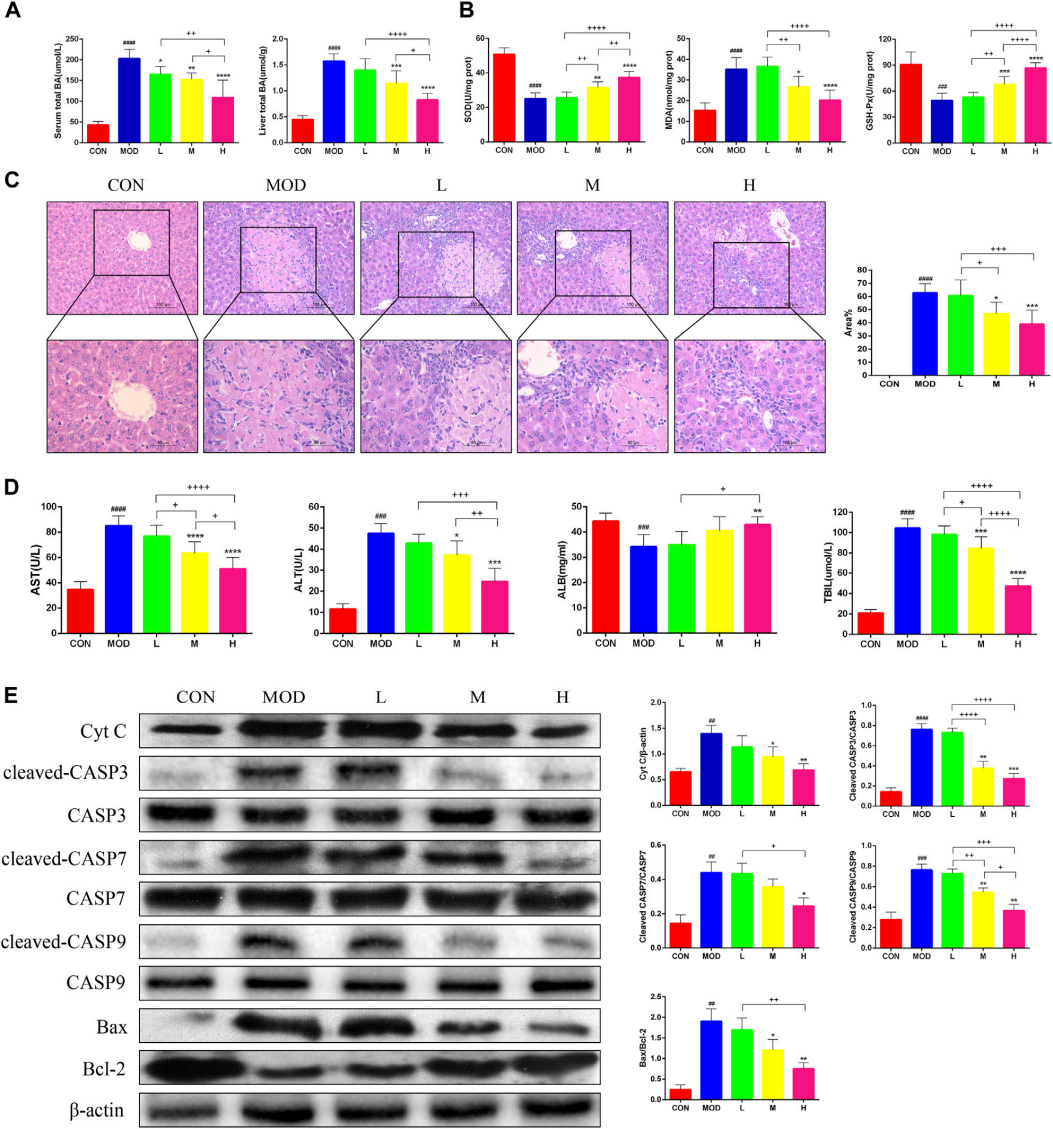
The effect of SAL on the oxidative stress and necrosis of hepatocytes caused by cholestasis. **(A)** Serum and liver total bile acid levels. **(B)** Indicators of liver oxidative stress. **(C)** H&E stain showing necrosis of hepatocytes. **(D)** Liver function indicators. **(E)** Western blot assay to detect the expression of apoptosis-related proteins in the liver. ^##^ *p* < 0.01, ^###^ *p* < 0.001, ^####^ *p* < 0.0001 VS. CON; *p < 0.05, **p < 0.01, ***p < 0.001 VS. MOD; ^+^ *p* < 0.05, ^++^ *p* < 0.01, ^+++^ *p* < 0.001, ^++++^ *p* < 0.0001. Data are expressed as mean ± SD.

The serum and liver TBA levels significantly increased in mice after bile duct ligation, and TBA was alleviated after SAL treatment (Figure 2A). The Superoxide Dismutase (SOD) and Glutathione Peroxidase (GSH-Px) levels in the liver were significantly lower and Malondialdehyde (MDA) levels were significantly higher in bile duct-ligated mice than in control mice, indicating that the deposition of TBA aggravated oxidative stress in the liver. After SAL treatment, the SOD and GSH-Px levels increased and the MDA level decreased (Figure 2B). Hematoxylin and eosin staining revealed extensive necrosis in the livers of mice in the model group, and the area of necrosis was reduced after SAL treatment (Figure 2C). The serum ALT, AST, and TBIL levels were significantly increased and the ALB level was decreased after bile duct ligation, whereas the concentration of ALB decreased, and we speculated that this might be caused by necrosis of hepatocytes due to BA stasis. We found that ALT, AST, and TBIL decreased and ALB increased after SAL treatment (Figure 2D). To verify the mechanism of hepatocyte necrosis, the expression of apoptosis-related proteins in the liver tissue was examined via western blotting. The cytochrome C (Cyt C) l, cleaved caspase-3 (CASP3), cleaved CASP7, cleaved CASP9, and Bax/Bcl-2 levels were significantly lower in the SAL-treated group than in the model group. The above experimental results suggest that SAL attenuates oxidative stress and hepatocyte necrosis caused by cholestasis.

### 3.3 SAL reduces liver fibrosis caused by cholestasis

**FIGURE 3:**
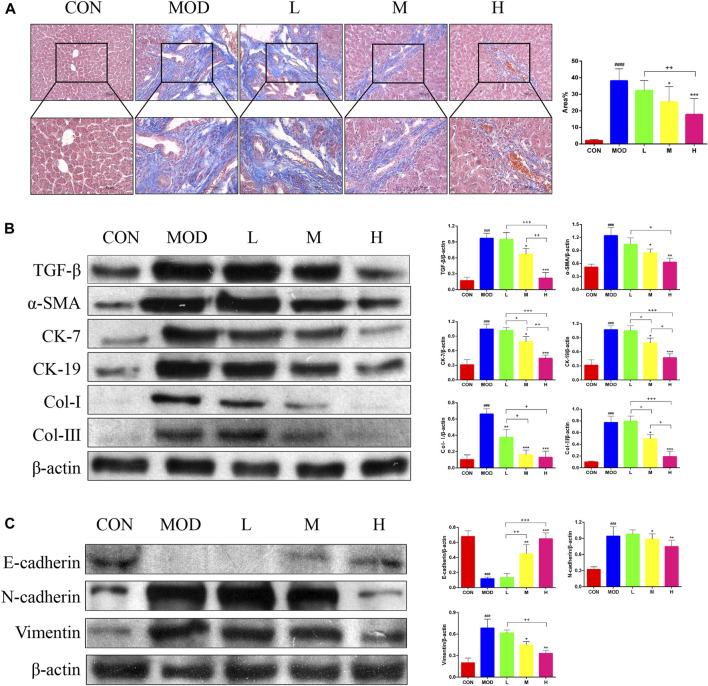
Effect of SAL on liver fibrosis caused by cholestasis. **(A)** Masson’s trichrome staining showing liver fibrosis. **(B)** Western blot assay to detect the expression of factors associated with liver fibrosis. **(C)** Western blot assay to detect EMT-related protein expression. ^###^ *p* < 0.001, ^####^ *p* < 0.0001 VS. CON; *p < 0.05, **p < 0.01, ***p < 0.001 VS. MOD; ^+^ *p* < 0.05, ^++^ *p* < 0.01, ^+++^ *p* < 0.001. Data are expressed as mean ± SD.

Masson’s trichrome staining of the liver tissue was performed to determine the effects of SAL on liver fibrosis. Masson’s trichome staining revealed a significant reduction in liver fibrosis after SAL treatment (Figure 3A). In addition, the results of western blotting experiments showed that the expression of TGF-β, α-SMA, Cytokeratin (CK)-7, CK-19, Collagen (Col)-Ⅰ, and Col-Ⅲ were significantly higher in the model group than in the control group, and the expressions of these proteins decreased after SAL treatment (Figure 3B). The expression of Epithelial-Mesenchymal Transition (EMT)-related proteins in the mouse liver was examined via western blotting. The epithelial phenotype of E-cadherin was reduced and the mesenchymal phenotypes of N-cadherin and vimentin were increased in the liver tissue of mice in the model group (Figure 3C). The above results suggest that SAL attenuates liver fibrosis caused by cholestasis.

### 3.4 SAL attenuates cholestasis-induced liver fibrosis *in vivo* by activating the PI3K/AKT/GSK-3β signaling pathway

**FIGURE 4:**
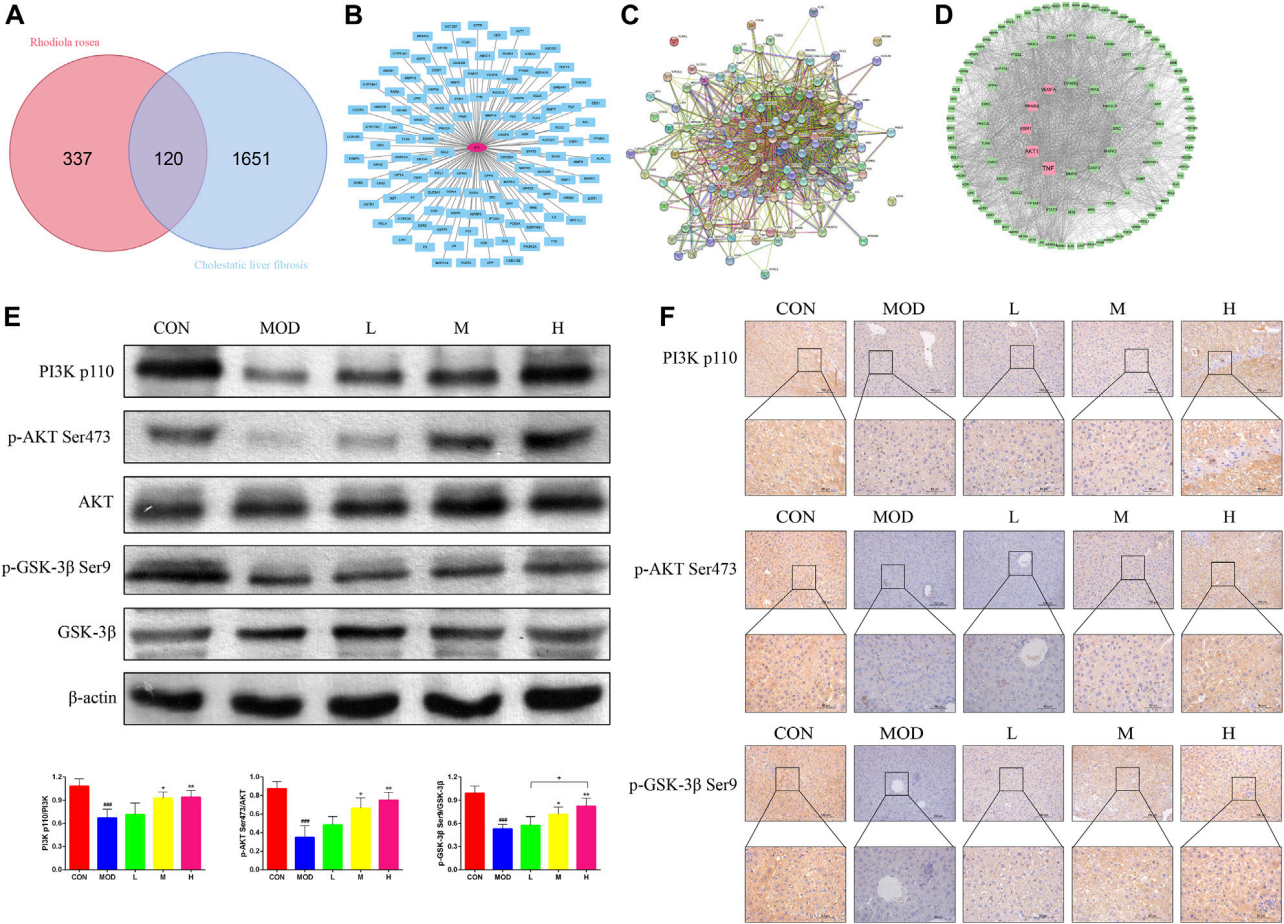
Effect of SAL on the PI3K/AKT/GSK-3β signaling pathway *in vivo*. **(A)** Venn diagram of SAL targets of action and cholestatic cirrhosis intersection genes. **(B)** Intersecting gene visualization processing. **(C)** Interaction analysis of the intersecting gene PPI protein network. **(D)** Core target screening. **(E)** Western blot detection of the effect of SAL on core proteins of the PI3K/AKT/GSK-3β signaling pathway. **(F)** Immunohistochemical detection of the effect of SAL on core proteins of the PI3K/AKT/GSK-3β signaling pathway. ^###^ *p* < 0.001 VS. CON; *p < 0.05, **p < 0.01 VS. MOD; ^+^ *p* < 0.05. Data are expressed as mean ± SD.

A total of 457 SAL targets (Supplementary Data S2) and 1771 genes related to the pathogenesis of cholestatic liver fibrosis (Supplementary Data S3), resulting in the identification of 120 intersecting genes (Figure 4A) and visualized (Figure 4B) and PPI protein network interactions (Figure 4C). The 120 genes were ranked according to their Betweenness centrality values. The top five genes were TNF, AKT, ESR1, PPARA, and VEGFA (Figure 4D). As AKT is a core gene of the PI3K/AKT signaling pathway, and GSK-3β is an important factor downstream of the PI3K/AKT signaling pathway, it was hypothesized that SAL exerts a protective effect against cholestatic liver fibrosis *in vivo* via the PI3K/AKT/GSK-3β signaling pathway. The expressions of the PI3K p110, p-AKT Ser473, and p-GSK-3β Ser9 proteins were significantly lower in the model group than in the control group for western blotting and immunohistochemistry. Gradual increases in the protein expressions of PI3K p110, p-AKT Ser473, and p-GSK-3β Ser9 were observed after treatment with SAL (Figures 4E, F). The above experimental results suggest that SAL acts *in vivo* by activating the PI3K/AKT/GSK-3β signaling pathway.

### 3.5 SAL inhibits HSC activation *in vitro* by activating the PI3K/AKT/GSK-3β pathway

**FIGURE 5:**
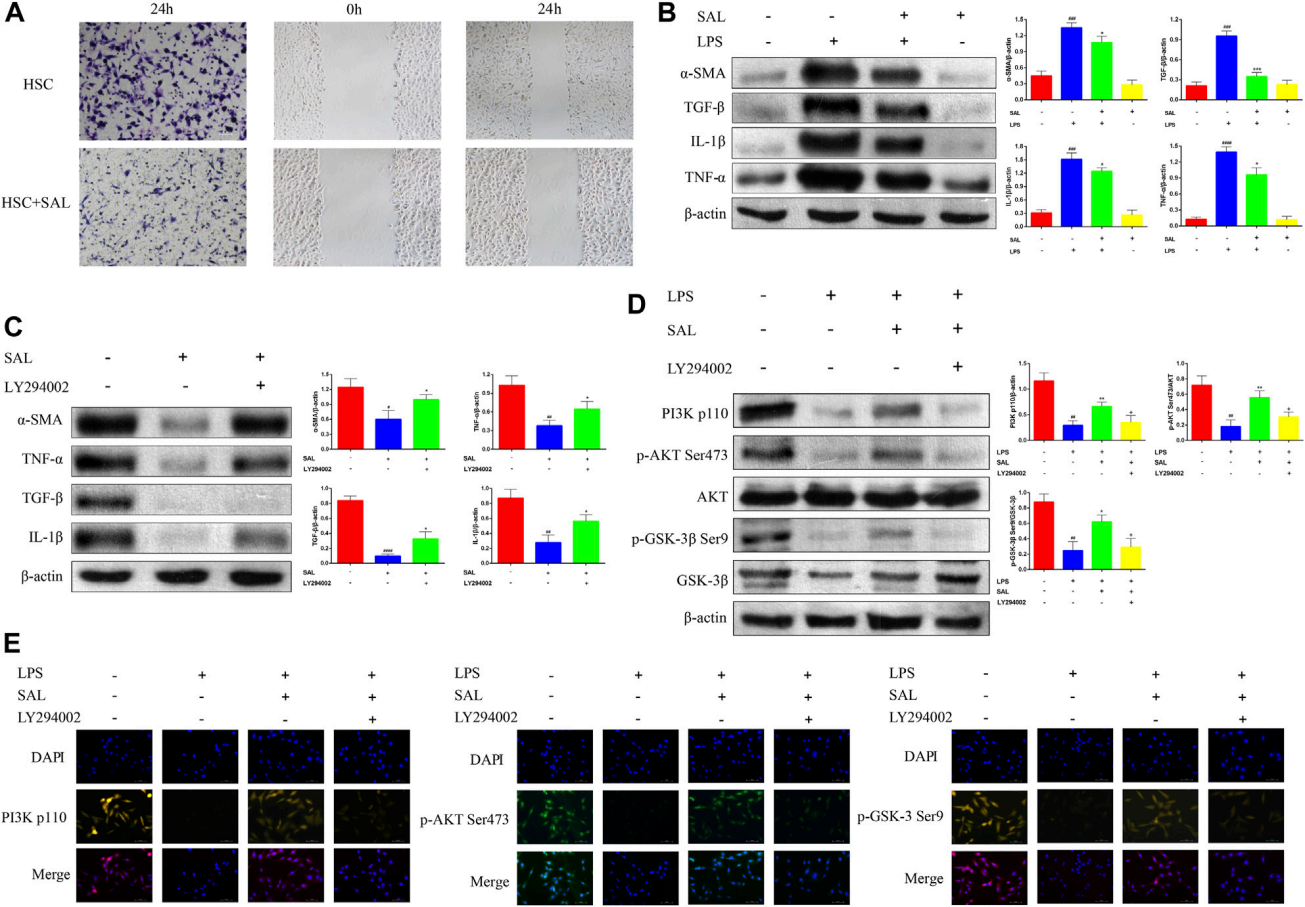
Effect of SAL on activated HSC. **(A)** Transwell and scratch assays to detect the effect of SAL on HSC invasion and migration. **(B)** Effect of SAL on the expression of HSC inflammatory factors. **(C)** Effect of SAL and LY294002 co-treatment on HSC inflammatory factors. **(D)** Western blot assay to detect the effect of SAL and LY294002 co-treatment on PI3K/AKT/GSK-3β signaling pathway. **(E)** Immunofluorescence detection of the effect of SAL and LY294002 co-treatment on the PI3K/AKT/GSK-3β signal transduction pathway. ^#^ *p* < 0.05, ^##^ *p* < 0.01, ^###^ *p* < 0.001, ^####^ *p* < 0.0001 VS. SAL (−)+LPS (−)/SAL (−)+LY294002 (−)/LPS (−)+SAL (−)+LY294002 (−);*p < 0.05,**p < 0.01,***p < 0.001VS.SAL (−)+LPS(+)/SAL (+)+LY294002 (−)/LPS(+)+SAL (−)+LY294002(−);^+^ *p* < 0.05 VS. LPS (+)+ SAL (+)+LY294002 (−). Data are expressed as mean ± SD.

SAL inhibited the ability of activated HSC to invade and migrate (Figure 5A). The expressions of the α-SMA, TGF-β, IL-1β, and TNF-α proteins were significantly increased in activated HSC and significantly decreased after SAL treatment (Figure 5B). Activated HSC were then co-treated with SAL and LY294002 (a PI3K inhibitor, abcam, UK). The expressions of the α-SMA, TGF-β, IL-1β, and TNF-α proteins were higher in co-treated HSC than in HSC treated with SAL alone (Figure 5C). Changes in the PI3K/AKT/GSK-3β signaling pathway after co-treatment were also investigated via western blotting assays. The expressions of PI3K p110, p-AKT Ser473, and p-GSK-3β Ser9 proteins were lower in co-treated HSC than in HSC treated with SAL alone (Figure 5D). The immunofluorescence results were consistent with the results of western blotting assays, and the fluorescence intensity of the co-treated group was attenuated compared to that of the group treated with SAL alone (Figure 5E). The above experimental results suggest that SAL acts *in vitro* by activating the PI3K/AKT/GSK-3β signaling pathway.

### 3.6 SAL regulates intestinal flora disorders caused by cholestasis

**FIGURE 6:**
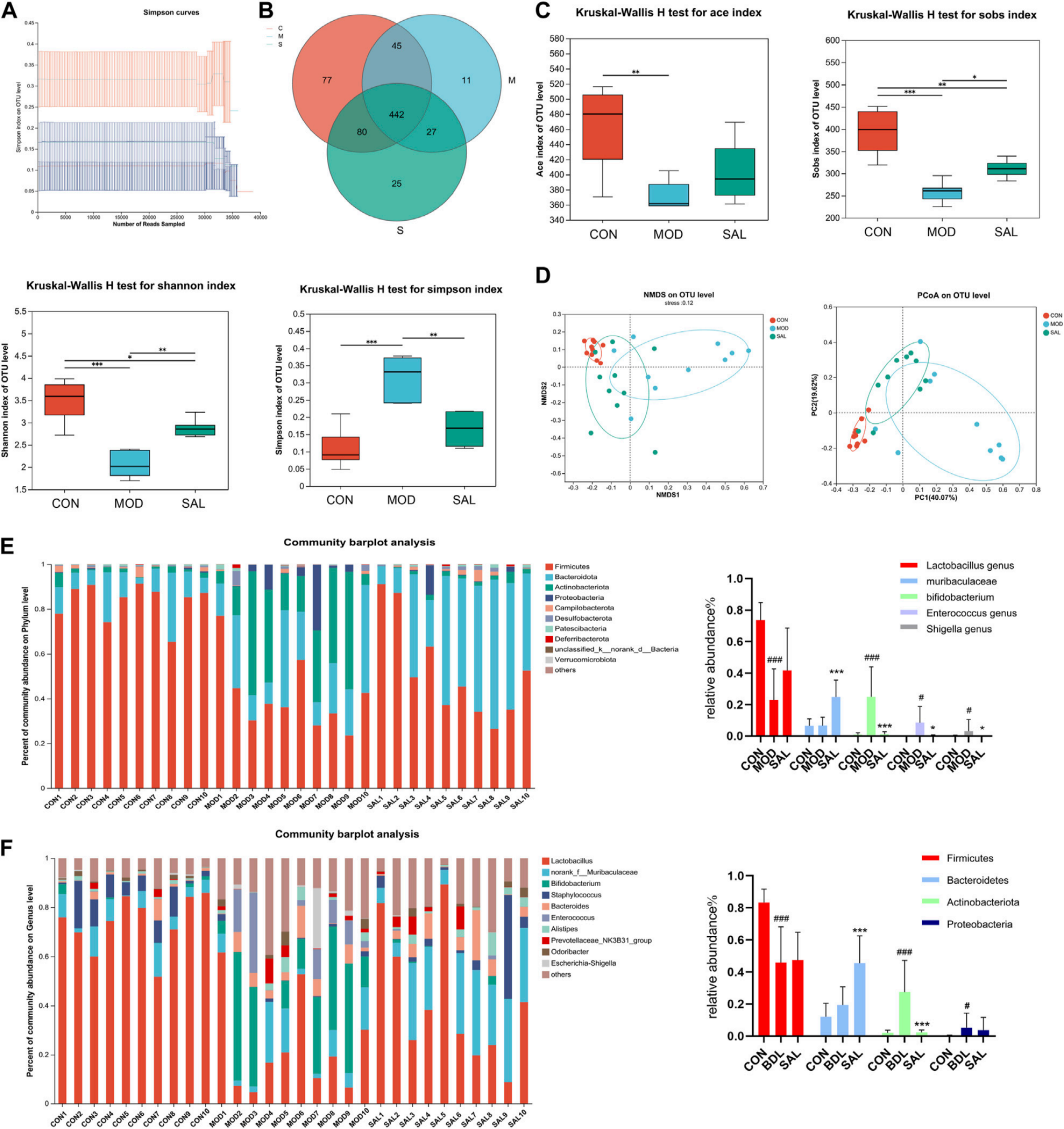
Effect of SAL on intestinal flora. **(A)** Sample dilution curves. **(B)** Venn diagram of species composition. **(C)** Alpha diversity analysis. **(D)** Beta diversity analysis. **(E)** Distribution of intestinal flora in each group of portal level. **(F)** Distribution of intestinal flora by groups at genus level. ^#^ *p* < 0.05, ^###^ *p* < 0.001 VS. CON; ^*^ *p* < 0.05, ^**^ *p* < 0.01, ^***^ *p* < 0.001 VS. MOD. Data are expressed as mean ± SD.

The intestine and liver are closely related anatomically and physiologically, and cholestasis may affect the intestine; therefore, mouse feces were used for intestinal flora testing. The dilution curves of the control, model, and high-dose treatment groups reached a plateau as the number of samples in each group increased, indicating the reliability of the results (Figure 6A). A total of 442 species (62.5%) were common to the control, model, and SAL treatment (62.51%), 77 species (10.89%) were unique to the control group, 11 species (1.55%) were unique to the model group, and 25 species (3.53%) were unique to the SAL treatment group (Figure 6B). The SAL treatment and control groups had 522 species (78.07%) in common, while the model and control groups had 487 species (68.88%). The results showed that the intestinal flora composition of bile duct ligation mice treated with SAL was closer to that of normal control mice. Ace, Sobs, and Shannon indices were significantly lower in the model group than in the control group, while Simpson’s index was significantly higher, suggesting that the diversity of intestinal flora in cholestatic mice is significantly reduced compared to the control group, after treatment with SAL, the Sobs and Shannon indices were significantly higher in the treatment group than those in the model group, and the Simpson index was significantly lower in the treatment group than in the model group (Figure 6C). Beta diversity analysis showed that the SAL treatment group was closer to the control group than to the model group (Figure 6D). This suggests that the microbiological composition of the intestinal tends to return to normal following treatment with SAL. The level of intestinal flora phylum showed that the composition of intestinal flora in the three groups of mice were Firmicutes, Bacteroidetes, Actinobacteria, and Proteobacteria. The model group had a significantly lower proportion of Firmicutes and a significantly higher proportion of Actinobacteria and Proteobacteria than the control group. After treatment with SAL, the relative abundance of Bacteroidetes significantly increased and the relative abundance of Actinobacteria significantly decreased (Figure 6E). At the genus level, the proportion of *Lactobacillus* significantly decreased and that of Bifidobacterium, *Enterococcus*, and Escherichia-Shigella significantly increased in the model group. The relative abundance of *Lactobacillus* was increased in the SAL-treated group compared to that in the model group, though the difference was not statistically significant. The proportion of Muribaculaceae significantly increased while that of Bifidobacterium, *Enterococcus*, and Escherichia-Shigella significantly decreased (Figure 6F). The above results suggest that SAL can repair the intestinal flora disorders caused by cholestasis.

### 3.7 SAL repairs the mechanical barrier protection of the intestinal mucosa

**FIGURE 7:**
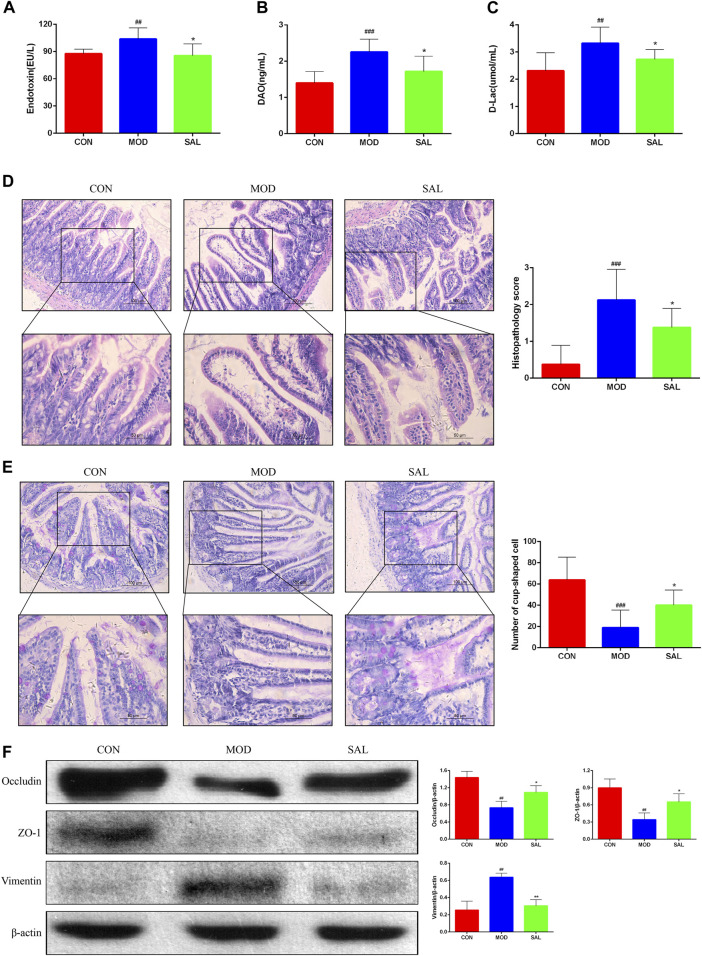
Effect of SAL on the mechanical barrier of intestinal mucosa. **(A)** Serum endotoxin concentration. **(B)** Serum diamine oxidase concentration. **(C)** Serum D-lactate concentration. **(D)** H&E stain showing histopathological changes. **(E)** PAS stain showing the number of cup cells in the intestinal mucosa. **(F)** Western blot assay for EMT-related protein expression. ^##^ *P*<0.01, ^###^ *p* < 0.001 VS. CON; *p < 0.05, **p < 0.01 VS. MOD. Data are expressed as mean ± SD.

The endotoxin diamine oxidase (DAO) and D-lactate levels were significantly higher in the model group than in the control group. The expressions of these genes were significantly decreased following SAL treatment (Figures 7A–C). Marked edema was histologically observed in the intestines of mice in the model group. In addition, the villi were shortened, small intestinal glands were reduced, alignment was disordered, and numbers of pannus and cup cells were reduced. However, these findings significantly improved after SAL treatment (Figures 7D,E). EMT disrupts the integrity of the intestinal mucosa, leading to increased ileal permeability and mucosal barrier dysfunction. Western blotting showed reduced expression of the epithelial phenotypes occludin and ZO-1 proteins and increased expression of the mesenchymal phenotype vimentin in intestinal tissues. After SAL treatment, the expressions of occludin and ZO-1 increased and that of vimentin decreased (Figure 7F). The above experimental results suggested that SAL can restore the protective effect of the intestinal mucosal barrier.

## 4 Discussion

Cholestasis, caused by biliary obstruction within and outside of the liver, induces the release of inflammatory cytokines and free oxygen radicals, resulting in hepatocellular damage, liver fibrosis, and liver failure. These changes may occur even if the obstruction is treated and the bile is cleared as the hepatocytes cannot be fully restored to their function in a short period of time and liver fibrosis may progressively develop. In this study, bile duct ligation was conducted to induce an animal model of extrahepatic cholestasis, targeting the PI3K/AKT/GSK-3β signaling pathway and intestinal flora, to investigate the therapeutic effect of SAL on obstructive cholestatic liver fibrosis and its mechanism. SAL effectively prevented and reversed hepatic fibrosis due to cholestasis by attenuating the inflammatory response and oxidative stress, reducing liver tissue necrosis, and inhibiting HSC activation and liver fibrosis. SAL also activated the PI3K/AKT/GSK-3β signaling pathway *in vitro* and *in vivo*. 16sRNA sequencing analysis revealed that SAL restored the abundance of intestinal flora and repaired damage to the intestinal mucosal barrier. These findings suggest that SAL ameliorates liver inflammation and oxidative stress caused by cholestasis through the activation of the PI3K/AKT/GSK-3β signaling pathway and inhibits the activation of HSC and liver fibrosis, suggesting that SAL is a potential therapeutic agent for cholestatic liver injury.

Sustained activation of HSC is a key component of the development of liver fibrosis. Under normal conditions, HSC exist in an inactive form and stimulation of the liver by excessive bile activates silent HSC to proliferate and aggravate liver fibrosis via enhanced intercellular signaling (Ezhilarasan, 2022). Therefore, HSC activation drives fibrosis. α-SMA is an important marker of HSC activation, and activated HSC produce large amounts of ECM (such as Col-Ⅰ and Col-Ⅲ) and inflammatory factors. TGF-β is an important pro-fibrotic signaling factor and mediates the EMT process (Shrestha et al., 2016). In addition, bile accumulation stimulates the proliferation of cholangiocytes (CK-7 and CK-19) to repair damaged hepatic tissues, further exacerbating liver fibrosis (Mariotti et al., 2018). In this study SAL reduced the release of inflammatory factors; inhibited HSC activation, proliferation, and migration ability; and reduced ECM deposition, alleviating the development of liver fibrosis. However, SAL may also attenuate the process of EMT, further alleviating hepatic fibrosis.

PI3K/AKT is one of the most important signaling pathways in organisms and is involved in the regulation of several life activities. PI3K is composed of a catalytic subunit, p110, and regulatory subunit, p85. PI3K p110 converts PIP2 to PIP3, which activates downstream effector molecules such as AKT. Complete AKT activation requires phosphorylation at Ser473 (Yang et al., 2020). After treatment with SAL, the PI3K p110 and p-AKT Ser473 expressions were upregulated, suggesting AKT activation. Activated AKT can inactivate GSK-3β via phosphorylation at the Ser9 site (Yang et al., 2020). GSK-3β is a PI3K/AKT downstream kinase involved in the development of a variety of disease states including inflammation, oxidative stress (Pecoraro et al., 2021), and fibrotic diseases (Wu X. et al., 2017). In the current study, phosphorylation of GSK-3β was attenuated upon BA sludge, which led to the activation of GSK-3β, promoting the activation and migration of HSCs and exacerbating the development of liver inflammation and fibrosis. SAL increased p-GSK-3β expression and inhibited cholestasis-induced GSK-3β activation, inhibiting HSC activation. The PI3K/AKT/GSK-3β signaling pathway is involved in the development of pulmonary fibrosis (Hu et al., 2022) and myocardial fibrosis (Liu et al., 2020). To determine if SAL is involved in the regulation of liver fibrosis through the PI3K/AKT/GSK-3β signaling pathway, LY294002, a PI3K inhibitor, was applied to HSC and effectively reversed the inhibitory effect of SAL on GSK-3β. These findings suggest that SAL participates in the regulation of HSC via activation of the PI3K/AKT/GSK-3β signaling pathway.

The pathogenesis of cholestasis is associated with imbalances in the microbiota of the intestinal flora. BA and the gut microbiota have a complex bidirectional relationship (Tian et al., 2020). BA produced in the liver effectively regulates gut microbial composition and gut barrier function, whereas gut microbial products also regulate hepatic BA synthesis. The disruption of the enterohepatic axis leads to the progression of most chronic liver diseases and inevitably leads to dysregulation of the intestinal microecology (Milosevic et al., 2019). Impaired bile secretion leads to a decrease in intestinal BA, which causes harmful flora to proliferate in the intestine, resulting in changes in the intestinal microbiological structure. Disturbances in the floral structure can lead to disruption of the intestinal mucosal barrier, resulting in increased intestinal permeability. Lipopolysaccharide (LPS) produced by gram-negative bacilli causes an intestinal inflammatory response, resulting in further damage to the intestinal mucosal barrier, creating a vicious cycle. The gut-liver axis is a bidirectional pattern of crosstalk between the intestine and the liver. Harmful microorganisms in the intestine enter the liver through the portal vein and stimulate hepatocytes to produce inflammatory factors that further activate HSC, leading to the development of cholestatic cirrhosis (Saha et al., 2022). In this study, SAL increased the abundance of Bacteroidetes and decreased the abundance of Actinobacteria at the phylum level. At the genus level, SAL reduced the abundance of harmful bacteria, such as *Escherichia*, *Shigella* and *Enterococcus*, in the intestinal tract and attenuated damage to the intestinal mucosal barrier. Therefore, SAL inhibits the release of LPS by reducing the abundance of harmful bacteria and acts as a protective barrier for the intestinal mucosa, reducing the entry of toxic substances into the liver via circulation, ultimately inhibiting the activation of HSC. However, in the absence of studies in gnotobiotic mice or adoptive fecal transfer from salidroside-treated mice to bile duct ligated mice, the microbiota analysis provides only correlative evidence for the role of changes in gut bacteria in protection against cholestasis-induced liver injury. Therefore, the specific mechanism of intestinal flora alteration in preventing cholestatic liver fibrosis should be further investigated.

Previous studies have found that SAL can attenuate CCL4-induced liver injury by inhibiting ROS deposition and restoring mitochondrial structure and function (Lin et al., 2019). SAL also inhibits the activity of NLRP3 inflammasome and reduces the release of inflammatory factors, thereby alleviating hepatic inflammatory injury (Qu et al., 2022). In addition previous studies have found that SAL inhibits L-glu-induced activation of JNK and MAKP, thereby inhibiting cytotoxicity and protecting brain tissue from toxic substances (Lee et al., 2013). In addition, SAL protects against diabetic kidney injury by activating the AKT/GSK-3β signaling pathway (Pei et al., 2022). SAL also attenuates myocardial ischaemia/reperfusion injury by inhibiting endoplasmic reticulum stress and mitochondrial fragmentation via AMPK signaling pathway (Tian et al., 2022). For lung tissue, SAL attenuates LPS-induced acute lung injury by inhibiting the TLR4/NF-κB signaling pathway (Jia et al., 2023).

We found that SAL was able to bind to AKT with minimum free energy through four hydrogen bonds, GLU-278, LYS-276, ASP-274, GLU-191 (Supplementary Figure S1) by molecular docking. Previous studies have identified that the PI3K/AKT/GSK-3β signaling pathway was associated with a variety of inflammatory diseases (Ma et al., 2018; Liu et al., 2019; Beltagy et al., 2024), therefore we suspected that SAL may activated the PI3K/AKT/GSK-3β signaling pathway. In this experiment, we found that SAL inhibited the progression of liver inflammation through activation of the PI3K/AKT/GSK-3β signaling pathway. It can reduce the stimulation of HSC, and inhibit HSC activation. On the other hand, we found that SAL can protect the intestinal mucosal barrier by adjusting the distribution of intestinal flora. This in turn reduces the number of toxic substances entering the liver via the “enterohepatic cycle” and ultimately inhibits the activation of HSC. Through both of these pathways, they can inhibit further liver lesions, reduces bile acid accumulation and oxidative stress, etc.

## Data Availability

The raw datasets presented in the study are deposited in the BioProject repository, available at, https://www.ncbi.nlm.nih.gov/sra/PRJNA1106253, accession number PRJNA1106253.
