# Supplementary figures and images for: Salidroside alleviates cholestasis-induced liver fibrosis by inhibiting hepatic stellate cells via activation of the PI3K/AKT/GSK-3β signaling pathway and regulating intestinal flora distribution
Source: Front Pharmacol. 2024 May 14;15:1396023. doi: 10.3389/fphar.2024.1396023 (PMC11130389; doi:10.3389/fphar.2024.1396023)

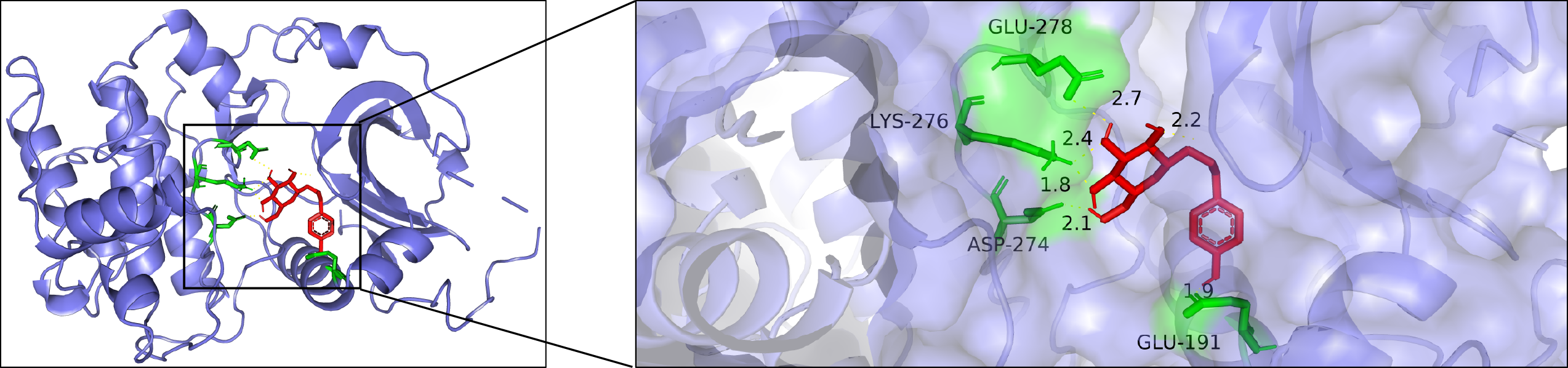

Supplement: Supplementary file 1 [file Image1.TIF]
