# Supplementary material for: Salidroside alleviates cholestasis-induced liver fibrosis by inhibiting hepatic stellate cells via activation of the PI3K/AKT/GSK-3β signaling pathway and regulating intestinal flora distribution
Source: Front Pharmacol. 2024 May 14;15:1396023. doi: 10.3389/fphar.2024.1396023 (PMC11130389; doi:10.3389/fphar.2024.1396023)
Supplement: Supplementary file 3 [file DataSheet1.docx]

Cytochrome C（#sc-13156 Texas, USA）、caspase3（#sc-7272 Texas, USA）、caspase3 p17（#sc-373730 Texas, USA）、caspase7（#sc-56063 Texas, USA）、cleaved-Caspase7（CST #9491 Massachusetts, USA）、caspase9（#sc-56073 Texas, USA）、cleaved-caspase9（CST #9505 Massachusetts, USA）、Bax（#sc-7480 Texas, USA）、Bcl-2（#sc-7382 Texas, USA）、PI3K p110（#sc-8010 Texas, USA）、AKT（#sc-56878 Texas, USA）、p-AKT（#sc-514032 Texas, USA）、GSK-3β（#sc-377213 Texas, USA）、p- GSK-3β（#sc-373800 Texas, USA）、TGF-β（#AF1027 China, Jiangsu）、α-SMA（#sc-32251 Texas, USA）、cytokeratin（CK）-7（#sc-23876 Texas, USA）、CK-19（#sc-376126 Texas, USA）、collagen Ⅰ（#sc-59772 Texas, USA）、collagen Ⅲ（#sc-271249 Texas, USA）、E-cadherin（#sc-8426 Texas, USA）、ZO-1（#sc-33725 Texas, USA）、N-cadherin（#sc-8424 Texas, USA）、Vimentin（#sc-6260 Texas, USA）、IL-1β（#sc-12742 Texas, USA）、TNF-α（#sc-12744 Texas, USA）、β-actin（#sc47778 Texas, USA）.
